# Ultrasensitive Photonic Microsystem Enabling Sub-micrometric Monitoring of Arterial Oscillations for Advanced Cardiovascular Studies

**DOI:** 10.3389/fphys.2019.00940

**Published:** 2019-07-23

**Authors:** Rosalía Rodríguez-Rodríguez, Tobias Nils Ackermann, Jose Antonio Plaza, Ulf Simonsen, Vladimir Matchkov, Andreu Llobera, Xavier Munoz-Berbel

**Affiliations:** ^1^Basic Sciences Department, Faculty of Medicine and Health Sciences, Universitat Internacional de Catalunya, Barcelona, Spain; ^2^Instituto de Microelectrónica de Barcelona (IMB-CNM, CSIC), Bellaterra, Spain; ^3^Department of Biomedicine, Aarhus University, Aarhus, Denmark

**Keywords:** photonic microsystem, integrated micro-optics, opto-mechanics, soft-lithography, myography, microvasculature

## Abstract

Cardiovascular diseases are the first cause of death globally. Their early diagnosis requires ultrasensitive tools enabling the detection of minor structural and functional alterations in small arteries. Such analyses have been traditionally performed with video imaging-based myographs, which helped to investigate the pathophysiology of the microvessels. Since new vascular questions have emerged, substantial modifications are necessary to improve the performance of imaging and tracking software, reducing the cost and minimizing the microvessel cleaning and manipulation. To address these limitations, we present a photonic microsystem fabricated in polydimethylsiloxane and integrating micro-optical elements and a lightguide-cantilever for sub-micrometric analysis of small arteries (between 125 and 400 μm of basal diameter). This technology enables simultaneous measurement of arterial distension, stiffness, vasomotion, and heartbeat and without the need for advanced imaging system. The microsystem has a limit of detection of 2 μm, five times lower than video imaging-based myographs, is two times more sensitive than them (0.5 μm/mmHg), reduces variability to half and doubles the linear range reported in these myographs. More importantly, it allows the analysis of intact arteries preserving the integrity and function of surrounding tissues. Assays can be conducted in three configurations according to the surrounding tissue: (i) isolated arteries (*in vitro*) where the surrounding tissue is partially removed, (ii) non-isolated arteries (*in vivo*) with surrounding tissue partially removed, and (iii) intact arteries *in vivo* preserving surrounding tissue as well as function and integrity. This technology represents a step forward in the prediction of cardiovascular risk.

## Introduction

Cardiovascular diseases (CVDs) are the first cause of death globally: more people die annually from CVDs than from any other cause (www.who.int)^1^. One of the major problems of these disorders is their late detection (Liu and Wang, 2016). Diagnosed CVDs are end-stages of a long-lasting pathological development that initiates downstream, where small arteries are located (Bosetti et al., 2016). Small blood vessels analysis has been for a long time limited to myographs (Mulvany and Aalkjaer, 1990; Buus et al., 2013), which developed to answer specific questions related to the regulation of vascular diameter in small-size arteries (Halpern et al., 1984). Despite widely used, most of these myographs are expensive, require camera-attached microscopes and advanced analysis software (Jadeja et al., 2015; Lawton et al., 2019). Even more importantly, these systems require an intense manipulation of the microvessel, which includes isolation from the body and removal of surrounding tissues, to be able to capture representative arterial diameter images. Some of new research questions are related to the bidirectional interaction of neighboring tissues (e.g., perivascular adipose tissue and arterial wall (Costa et al., 2018). These new questions require modifications of the original systems as well as substantial variations in the tools used to measure artery diameter and additional properties of the blood vessel (Lawton et al., 2019). Some interesting contributions have been recently performed, including intravital microscopy combined to laser speckle imaging (Nyvad et al., 2017) and advanced tracking software, such as VasoTracker (Lawton et al., 2019). Unfortunately, even considering these advantages, it becomes increasingly accepted that the early detection of arterial disorders before clinical conditions are manifested requires new specific strategies and tools for sensitive analysis of small artery structure and function.

In the base of the latter, we present a photonic microsystem fabricated in the low cost elastomer polydimethylsiloxane (PDMS) and integrating micro-optic elements and a lightguide-cantilever for ultrasensitive analysis of intact small arteries *in vivo*. In the lightguide-cantilever, light is coupled and guided through the cantilever until reaching the tip, which is aligned with the collecting optical fiber. The bending of the cantilever in response to transversal mechanical loads (Llobera et al., 2009) or forces (de Pedro et al., 2014a,b) misaligns the tip and the collecting fiber, increasing the optical losses of the photonic microsystem. This displacement and the corresponding optical losses are proportional to the magnitude of the mechanical loads/forces and can be used to monitor in real time the forces applied to the cantilever. Since intact small arteries are in close physical contact with the cantilever, the photonic microsystem responses to mechanical loads associated to arterial processes. Such processes include arterial distension, stiffness, vasomotion, and even the small amplitude arterial oscillations corresponding to heartbeat.

We validated the mechanical performance of the photonic microsystem *in vitro* in isolated rat small mesenteric arteries pressurized in myographs. We investigated then the mechanical performance of this system during drug-induced vasodilatation/vasoconstriction responses *in vivo* in small arteries of living anesthetized rats: first removing the surrounding tissues for comparison with the imaging systems and finally using intact microvessels. The photonic microsystem enabled real time evaluation of mechanic performance of the microvasculature *in vivo*. The objective of this ultrasensitive laboratory tool is to identify patterns enabling to understand the pathophysiological routes leading to CVDs.

## Results and Discussion

### Design and Simulation of the Lightguide-Cantilever

**Figure 1:**
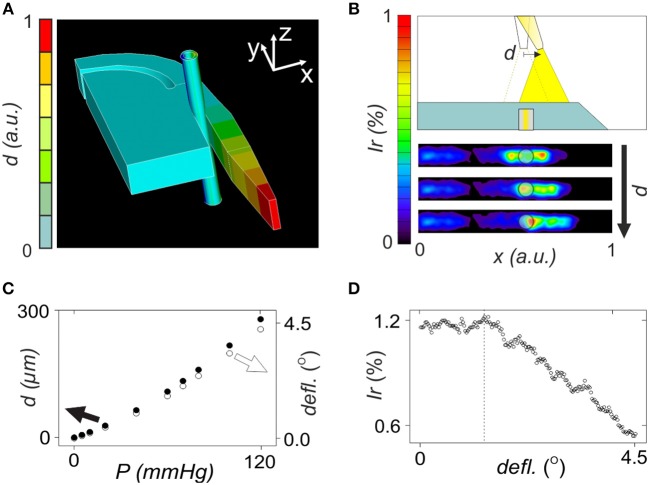
Numerical evaluation of opto-mechanical performance of the lightguide-cantilever. **(A)** Image of the mechanical response of the 3D model of the microsystem after exposure of the blood vessel to 120 mmHg intraluminal pressure. **(B)** Representation of a variation in deflection angle and displacement with the applied intraluminal pressure according to ANSYS numerical analysis, when considering the model presented in **(A)**. **(C)** Performance of the optomechanical sensing of the microsystem with the tip of the lightguide-cantilever displacing to the right (black arrow) in response to mechanical forces. Figure also includes irradiance maps corresponding to the detection surface of the microsystem at three increasing deflection angles, i.e., 1, 2, and 3°. The semi-transparent circle represents the optical fiber connected to the detector, coinciding with that in size and position in the microsystem. **(D)** Representation of a variation in irradiance percentage with the deflection angle, according to the values obtained by TracePro when considering the model presented in **(C)**.

**Figure 2:**
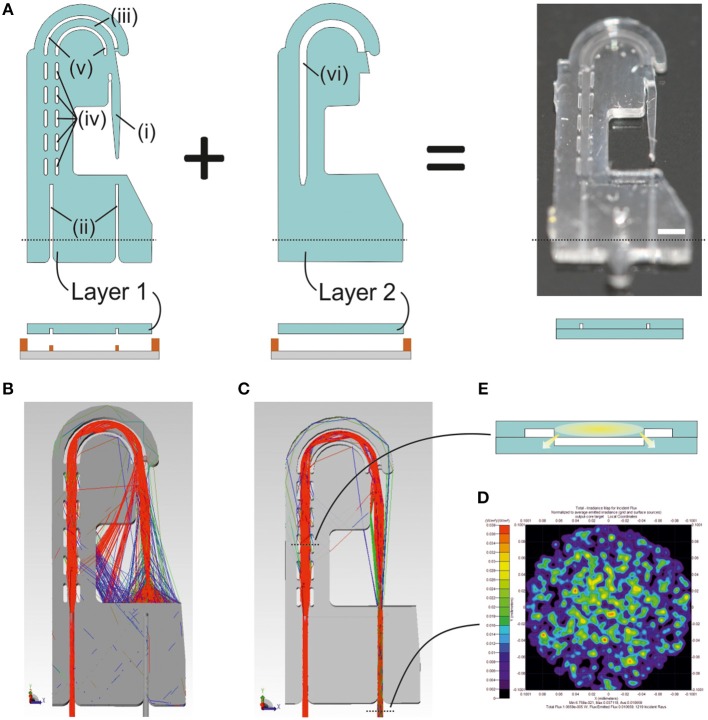
Design and fabrication of the photonic microsystem. **(A)** Illustration of the design and the fabrication scheme for two-layer photonic microsystem, including (i) the lightguide-cantilever, (ii) the self-alignment elements, (iii) the PDMS rib-waveguide, (iv) the curved, (v) straight air cavities, and (vi) the air cavity in the second layer aligned with the ones in the first layer. The black dotted line in the image corresponds to the transversal section of the design illustrated in the fabrication protocol. An image of the final microsystem includes a white line corresponding to the scale of 1 mm. **(B)** Ray tracing simulations of a 3D model of the photonic microsystem illustrating the light that, after being coupled into the device, is confined into the lightguide-cantilever. The simulation shows the wide aperture angle of the lightguide-cantilever, fundamental for the sensing capacity of the system. **(C)** Ray tracing simulations of a 3D model of the photonic microsystem, this time illustrating only these rays that, after being coupled into the system, reach the collection optical fiber and the detector. Two black dotted lines are present in the image. The bottom one indicates the region were the **(D)** irradiance map of the light confined in the out-coupling optical fiber later reaching the detector is acquired. The top dotted line corresponds to the transversal section of the microsystem, illustrated in **(E)**. This transversal section shows the geometry of the leaky rib-like element designed to transport the light from the in-coupling area to the lightguide-cantilever. The leaky regions of the lightguide, generated by the direct contact between PDMS structures at the edges of the microsystem, are indicated in the image.

The optical microsystem was designed to analyze small artery function and mechanical properties. To this end, it includes a region to hook on small arteries with a free-moving lightguide-cantilever in physical contact with perivascular tissues (Figures 1, 2). The lightguide-cantilever is the core element of the photonic microsystem. This element is conceived to deflect in response to mechanical loads associated to arterial structure and function. Briefly, when arterial diameter is changed (e.g., dilation or contraction), it proportionally transduces to displacement of the lightguide-cantilever. This displacement modifies the amount of light collected in the detection area. Since the collection optical fiber connected to the detector was initially aligned with the lightguide-cantilever (when in basal conditions), the displacement misaligned the cantilever with the collecting fiber, producing a change in the amount of collected light. Thus, the amount of collected light is proportional to the misalignment of the cantilever, and to the diameter change. It is possible in part because of the mechanical properties of PDMS, the material used in fabrication of the photonic microsystem. PDMS has a Young's modulus similar to the parameters reported for biological tissues. This minimizes the interference with mechanical responses and facilitates the transferences of the mechanical loads from the vascular wall to the cantilever.

The numerical evaluation of the cantilever performance was conducted in separate by opto-mechanical and optical analysis as described below.

In opto-mechanical analysis, the displacement of the cantilever in response to mechanical loads was studied (Figure 1). A 250 μm cylinder with an inner diameter of 150 μm emulated the artery, which was positioned in the region between the cantilever and the bulk. Mechanical properties of this structure were defined according to bibliography to resemble those of microvessels (Izzard et al., 2005; Shimokawa and Satoh, 2014). Increasing mechanical loads were applied inside the artery simulating an increase of intraluminal pressure from 0 to 120 mmHg, which was in accordance to experimental myography assays with arterial segments (Rodríguez-Rodríguez et al., 2009; Ogalla et al., 2015). In mechanical simulation, the increase of the intraluminal pressure produced a progressive deflection of the lightguide-cantilever (see video in the Supplementary Information S1). This displacement was more pronounced in the cantilever's tip (Figure 1A) and its magnitude (or the deflection angle) correlated to the applied intraluminal pressure over the range of study (Figure 1B). The theoretical sensitivity of the system was 2 μm/mmHg of applied pressure.

Optical ray tracing simulations were performed to correlate previous displacements with optical losses in the microsystem by the misalignment between the cantilever's tip and the collecting optical fiber. In the model, the deflection angle was varied according to mechanical simulations and the amount of light reaching the detector was determined (examples of ray tracing simulations at different deflection angles are in Supplementary Information S2). The deflection of the cantilever displaced the light cone outgoing from the cantilever's tip (Figure 1C), reducing the amount of light reaching the light output optical fiber and the detector. In simulations, optical losses associated to the displacement of the cantilever were proportional to the deflection angle and thus to the applied intraluminal pressure in the range of study (Figure 1D). The large numerical aperture of the lightguide-cantilever may be the responsible to confer the microsystem with this wide sensing range. According to simulations, the microsystem should be able to detect diameter changes associated to the variation of intraluminal pressure of around 2 μm. These simulations validated the sensing principle of the lightguide-cantilever.

### Photonic Microsystem for Ultrasensitive Small Arteries Analysis

Figure 2 illustrates the design and fabrication of the photonic microsystem. The microsystem integrated micro-optical elements facilitating in-coupling, guidance and out-coupling of light. The design consisted of two independent layers, aligned and bonded to build the final structure of the microsystem (Figure 2A). The first layer contained the (i) lightguide-cantilever, (ii) self-alignment elements for fiber-optics positioning (Muñoz-Berbel et al., 2013), and (iii) an U-shaped PDMS lightguide defined by a set of (iv) parallel and (v) curved air cavities acting as mirrors to guide the coupled light from the inlet fiber-optics to the cantilever. The U-shaped architecture of the lightguide increased optical losses but allowed the positioning of both light inlet and outlet at the same region of the system, simplifying microsystem implementation *in vivo*. These elements were sealed with a second layer, which only contained (vi) an air cavity-mirror aligned to the U-shaped PDMS structure from the first layer for transversal light confinement, thus resembling the architecture of a rib-waveguide (Doylend et al., 2011). The second layer sealed the structure and prevented the filling of the air cavities with liquid during measurements.

Light coupling and decoupling was performed through optical fibers connected to external light sources and spectrometers. This conferred the microsystem with high flexibility and adaptability, being possible to change light source and detectors even along the experiments.

Ray tracing simulations of the microsystem (Figure 2B) confirmed that total internal reflections at the PDMS-air interfaces allowed light injected by an optical fiber to be guided along the rib-like structure and coupled into the lightguide-cantilever (a complete description of the performance of air mirrors is in Supplementary Information S3). At the cantilever's tip, the outgoing cone of light presented high numerical aperture due to the large difference between the refractive indexes of PDMS and air. Less than 20% of the light confined in the waveguide was finally collected by a second optical fiber positioned in front of the cantilever's tip (Figure 2C). This was one of the reasons for the low coupling efficiency of the microsystem, which was theoretically of around 1% from the total power injected (Figure 2D). Other design factors that affected light coupling efficiency were: the curvature of the PDMS lightguide, necessary to minimize the amount of un-coupled light reaching the detector, to enhance the signal-to-noise ratio and to simplify microsystem implementation; the leaky nature of the rib-waveguide, since the rib structure was lost at the edge (Figure 2E); and the use of a parallel set of air cavities instead of a large continuous air cavity, which was necessary to guarantee the mechanical integrity of the microsystem. It should be note that most of the optical losses were due to the wide angular aperture of the microsystem at the cantilever's tip, which, on the other hand, contributed to expand the dynamic measurement range of the microsystem. That is, a large aperture angle in the cantilever's tip enlarged the analytical range of the microsystem, which should be able to distinguish larger deflections.

### *In vitro* Recordings in Small Mesentery Rat Arteries

Vascular responses to luminal pressure changes and drugs are often used to determine structural and functional properties of small arteries *in vitro*, providing good correlation with cardiovascular risk (Rizzoni et al., 2009). These assays are currently performed with pressure myographs, here employed as golden standard technique in the validation of the microsystem.

**Figure 3:**
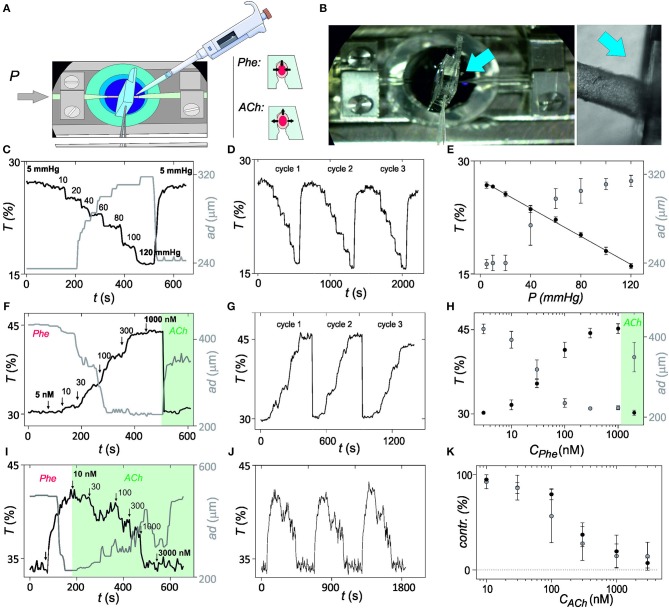
*In vitro* validation of optical myography. **(A)** Illustration of the *in vitro* set-up and protocol for validation of the mechanical sensing with the photonic microsystem. Microsystem is implemented in the pressure myograph and properties of the microvessel are evaluated after intraluminal pressure changes or after addition of vasoconstrictor phenylephrine (Phe) or vasodilator acetylcholine (ACh) at 70 mmHg intraluminal pressure. **(B)** Image of the *in vitro* set-up, with a magnification in the region where microvessel and microsystem have physical contact (blue arrow indicates the region). **(C)** Representation of the variation of light transmittance at 700 nm (*T*, from photonic microsystem) and arterial diameter (*ad*, from videomicroscope) over time after exposure to intraluminal pressure steps from 5 to 120 mmHg. **(D)** Variation of the optical myography recordings over time after application of three consecutive intraluminal pressure cycles. **(E)** Averaged results for changes in light transmittance and outer diameter magnitude with the intraluminal pressure with the applied pressure steps shown in **(C)**. **(F)** Light transmittance and outer diameter changes in response to Phe (5 nM−1 μM). **(G)** Optical myography recordings over time after three consecutive Phe cycles. **(H)** Averaged changes in light transmittance and outer diameter with Phe application shown in **(F)**. **(I)** Changes in light transmittance and outer diameter in response to ACh (10 nM−3 μM) in Phe-constricted artery. **(J)** Optical myography recordings over time after three consecutive Phe-ACh cycles. **(K)** Averaged and normalized results from the experiment **(I)**. Error bars represent standard deviation (95% confidence) (*n* = 5).

Figure 3A describes the experimental *in vitro* protocol. Intraluminal pressure changes and vascular response to phenylephrine (Phe) and acetylcholine (ACh) were used to validate the microsystem. The microsystem was implemented in the myograph as illustrated in Figure 3B and dilation/contraction processes were recorded simultaneously with both techniques, acquisition camera or photonic microsystem. The myography validation consisted of three assays to: (1) determine the structural integrity of the vessel, (2) evaluate the functional integrity of the vessel, and (3) determine endothelial function. Considering the latter, the dysfunction of the endothelium, i.e., the inner cell layer in blood vessels, is reported in many CVDs, such as hypertension (Deanfield et al., 2007) or diabetes (Briones et al., 2014). It is important to mention that the analytical properties presented below are only used for comparison of both imaging-based pressure myograph and optical microsystem and should not be considered in general since they may depend on the characteristics of the microvessel and the experimental conditions.

In the first assay, structural integrity was determined by progressively increasing intraluminal pressure from 5 to 120 mmHg and subsequent recovery to the initial pressure values. Three consecutive pressure cycles were performed. The gradual dilatation and subsequent relaxation of the microvessel was recorded with the videomicroscope in the pressure myograph and with the microsystem by monitoring transmitted light at 700 nm, which called optical myography (Figure 3C; continuous optical myography recording in Figure 3D). First difference between transduction methods was in the detection limit. Whereas, under the current experimental conditions, myography required an applied pressure of almost 20 mmHg to detect a change (limit of detection = 17 mmHg from 3σ method) associated to an arterial dilation of 10 μm, the photonic microsystem detect differences even below 10 mmHg (limit of detection = 3 mmHg from 3σ method), which corresponded to an arterial diameter change around 2 μm. Also the dynamic range of analysis and the response of both systems was different. By video imaging-based myography, intraluminal pressure changes from 80 to 120 mmHg were indistinguishable and the linear dynamic range was thus restricted to magnitudes between 20 and 60 mmHg. The photonic microsystem improved pressure myograph almost doubling the dynamic measurement range (from 5 to 120 mmHg; Figure 3E) and reducing to half the measurement variability (average coefficient of variation of 1.7 vs. 3.0% obtained with the myograph). The sensitivity of the optical myography was of 0.5 μm/mmHg of applied pressure, close to the 2 μm/mmHg theoretically predicted, and two times more sensitive than myography (1.1 μm/mmHg). It should be emphasized that this proportionality between transmittance and intraluminal pressure in a viscoelastic system, such as the artery may be only possible to find *in vitro*. In this situation, the biological system may present long time constants, quasi-static, thus having enough time to adapt and to behave as it was elastic.

In the second assay, the functional integrity of the artery was evaluated by exposure to alpha-adrenergic agonist Phe and subsequent relaxation with ACh. Cumulative application of Phe from 5 to 1,000 nM successively constricted the microvessel. Similar records were obtained with both techniques (Figure 3F; temporal optical myography recording in Figure 3G) with small discrepancies in sensitivity, variability, and reversibility (Figure 3H). As before, the microsystem was more sensitive, expanded the linear range in the region of high Phe concentrations and presented lower variabilities. The time necessary to recover the basal level after relaxation was also much smaller with the microsystem. These small improvements may be attributed to a divergence between arterial diameter and stiffness. Cumulative Phe assays performed in arterial segments before and after the implementation of the microsystem provided comparable responses (Supplementary Information S4), confirming that the photonic microsystem was not compromising the integrity or functionality of the vessel.

As a third assay, endothelial response was evaluated by exposure of pre-constricted arterial segments to the endothelium-dependent vasodilator ACh. The cumulative application of ACh between 10 and 3,000 nM produced a concentration-dependent arterial relaxation, providing comparable results from both techniques (Figure 3I; temporal optical myography records in Figure 3J). A certain oscillation of the arterial diameter was observed in both cases, although with more resolution with the microsystem, probably due to vasomotion (Rahman et al., 2007; Aalkjær et al., 2011). Comparing the averaged values from triplicates, it is clear that both techniques provided comparable results, although the microsystem was again more sensitive to arterial diameter changes and presented lower variability (Figure 3K). Similar behaviors were obtained with arterial segments before and after the implementation of the microsystem (Supplementary Information S5), confirming that the microsystem was not affecting endothelial function.

### *In vivo* Recordings in Small Mesentery Rat Arteries

The photonic microsystem allowed functional analysis *in vitro*, surpassing conventional video imaging-based pressure myograph. However, the displacement *in vitro* by artificial mechano-fluidic pressure changes of the liquid does not represent reality. *In vivo* pressure changes are caused by a number of control mechanisms, including arterial wall biochemical/physiological properties, the contractile state of smooth muscle cells and the flow mediated activity of endothelium, among others. Hence, *in vivo* results may vary from those obtained *in vitro*.

**Figure 4:**
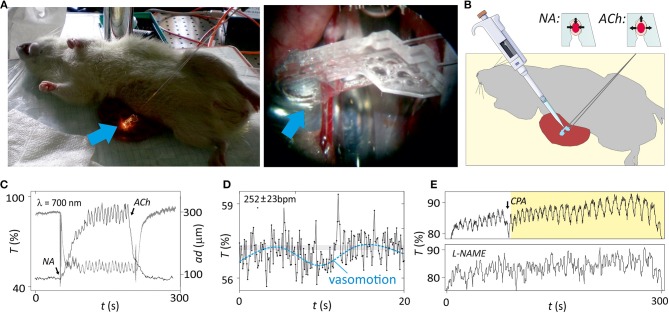
*In vivo* analysis of isolated microvessels. **(A)** Image of the *in vivo* set-up, with a magnification in the region where microvessel and microsystem are in a physical contact (blue arrow indicates the region). **(B)** Illustration of the *in vivo* set-up and protocol for analysis of structure and function of the microvessel with the microsystem. Functional properties of the microvessel are evaluated assessing vasoconstriction with noradrenaline (NA) or vasodilatation with acetylcholine (ACh). A drawing depicted in this figure was obtained from the image repository Smart Servier Medical Art (https://smart.servier.com/). **(C)** Changes in light transmittance at 700 nm (T) and arterial diameter (ad, from videomicroscope) over time after application of 5 μM NA and the following application of 5 μM ACh. Periodic oscillations corresponding to vasomotion were clearly observed during contraction. **(D)** Magnification (×15) plot the region target in **(C)** illustrating the cantilever deflection caused by heartbeat and vasomotion (dash line). The average heart rate is calculated as the number of oscillations (beats) per minute. **(E)** Modulation of the vasomotion by addition of cyclopiazonic acid (CPA) or L-NG-Nitroarginine methyl ester (L-NAME) (*n* = 5).

The photonic microsystem was implemented in small mesenteric arteries of anesthetized rats as illustrated in Figure 4A. All tissues surrounding the microvessel, i.e., connective tissue and fat, were removed before implementation to be able to compare with videomicroscopy records. The experimental protocol involved vasoconstriction with the alpha-adrenergic agonist noradrenaline (NA) and relaxation to ACh (Figure 4B). NA was used instead of Phe for presenting an analog activity and being already optimized in *in vivo* assays. Optical myography was recorded at a wavelength of 700 nm to avoid interference of absorbing species and compared to videomicroscopy (Figure 4C). With both technologies, comparable contraction and dilation processes were observed, with oscillation patters in contracted arteries associated to vasomotion (Figures 4C,D). This time, however, the oscillation pattern was clearer than *in vitro* since the microsystem was operating at lower integration times.

Vasomotion is important for diagnosis since alterations of this oscillatory pattern have been observed in patients suffering from diabetes or hypertension (Aalkjær et al., 2011). The mechanism for vasomotion is not fully understood but some evidences show that vasomotion in mesenteric arteries may depend on endothelium-derived NO production (Peng et al., 2001; Nyvad et al., 2017). Accordingly, the microsystem detected inhibition of vasomotion after incubation of the artery with L-NAME (Figure 4E), while drug-mediated arterial distension was not affected. Moreover, it was previously found that an interplay with different oscillators in the vascular wall could affect the vasomotion pattern (Rahman et al., 2007). Thus, inhibition of intracellular oscillator by emptying the sarcoplasmic reticulum by exposure of the vascular wall to cyclopiazonic acid (CPA) leaded to switch from the conventional vasomotion pattern oscillations to large amplitude and low frequency. This CPA-induced vasomotion was not NO-dependent. Instead, it was a product of endothelium and smooth muscle membrane oscillators interacting via the current running between the two cell types and setting up anti-phase oscillations in calcium (Rahman et al., 2007). Optical myography recording with the photonic microsystem confirmed the CPA-induced switch of vasomotion type *in vivo* with unchanged parameters of other oscillations (Figure 4E).

Additionally, the magnification of microsystem measurements showed a third frequency pattern of small amplitude that could be associated to the heartbeat of the animal (Figure 4D; a video with the lightguide-cantilever oscillation associated to this process is in Supplementary Information S6). Proper heartbeat registers required low integration times below 150 ms (optimization in Supplementary Information S7) since above this value the sampling rate was too close to the heartbeat frequency. The heartbeat, therefore, was only appreciable when small integration times were used, but the measurement of this small amplitude frequency pattern demonstrated the high sensitivity and resolution of the photonic microsystem.

### Evaluation of the Bidirectional Interaction Between Arteries and Perivascular Tissues in Intact Rat Mesenteric Arteries *in vivo*

The study of small size arteries from relevant microvascular beds, such as the mesentery requires important manipulation of the vessel, either the section and isolation of arterial segments (e.g., wire myography) (Rodriguez-Rodriguez et al., 2008; Heagerty et al., 2010) or the removal of most of fat and connective tissue (e.g., pressure myography) (Shahid and Buys, 2013; Jadeja et al., 2015). It represents an important risk of damage or alteration of arterial function and the impossibility to evaluate the bidirectional interaction between tissues.

**Figure 5:**
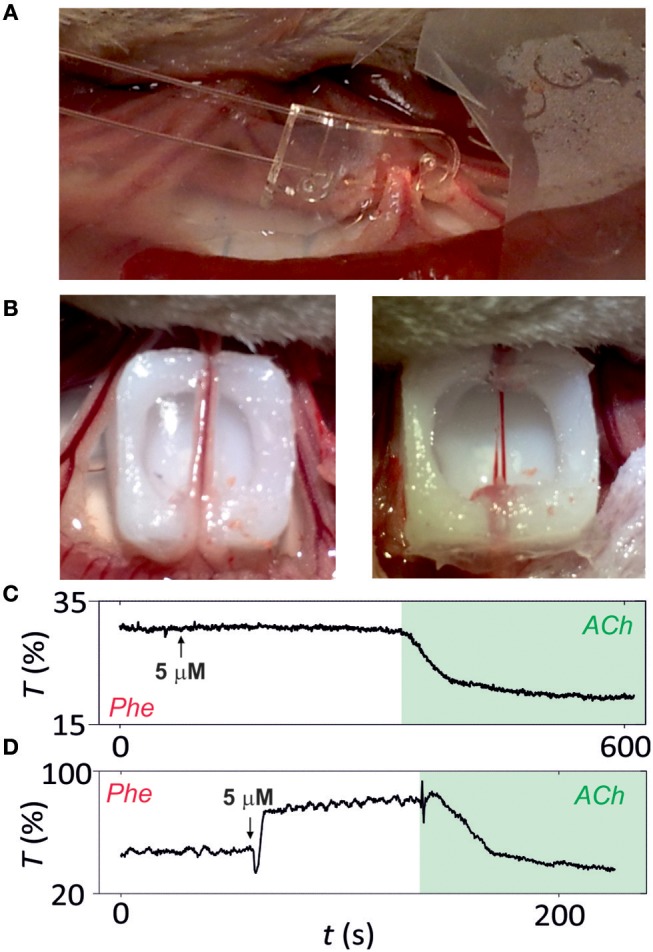
Analysis of intact microvessels *in vivo*. **(A)** Image of microsystem implemented in intact microvessels. **(B)** Image of the microsvessel before (left) and after (right) fat and connective tissue removal. Functional analysis of **(C)** intact and **(D)** isolated microvessels (after surrounding tissue removal) by vasoconstriction with noradrenaline (NA) or vasodilatation with acetylcholine (ACh). Microvessel function is assessed by following transmittance variation (in percentage) over time (*n* = 5).

The photonic microsystem surpasses the performance of wire and pressure myographs by enabling the analysis of intact microvessels from rat mesentery with minimal vessel manipulation (Figure 5A). Microvessels before and after the removal of perivascular fat and connective tissue were analyzed with the microsystem (Figure 5B). Vasoconstriction with the alpha-adrenergic agonist NA and relaxation with ACh was used to evaluate arterial function. Figures 5C,D illustrate the response of the vessel with and without surrounding tissues, respectively. When surrounded by fat and connective tissue, the vessel presented a very small contraction in response to NA but a large relaxation with ACh and contracted artery did not present vasomotion (Figure 5C). In opposition, when removing the surrounding tissues, the microvessel showed a symmetric contraction-relaxation process, as obtained in previous experiments with isolated segments, and presented vasomotion, especially when contracted (Figure 5D). This result confirmed that the tissues surrounding the vessel might not only be structural but presenting functionality, concretely an important vessel pre-constriction.

## Methods

### Design, Simulation, and Fabrication Protocol

AutoCAD (Autodesk, California, US) was used for the design of the photonic microsystem. A 3D model of the design was analyzed with mechanical and optical simulation software. Mechanical simulations were performed by Finite Element Method (FEM) using ANSYS® Multiphysics (Release 14.5, http://www.ansys.com). The mechanical performance was modeled using the 3D element SOLID95, a higher-order element defined by 20 nodes, which were well-suited to model curved boundaries. Large-deflection calculations were also activated when required. The Young's Modulus of the microsystem and the microvessel were set according to the bibliography to 800 kPa (Fuard et al., 2008) and 450 kPa (O'Rourke et al., 2002), respectively. In mechanical simulations, the deflection of the lightguide-cantilever in response to increasing pressures in the microvessel, i.e., from 0 to 120 mmHg, was evaluated.

TracePro simulation software (Release 7.8, Lambda Research, Littleton, MA, USA) was used for optical simulations. The optical properties of each element in the 3D model were set according to bibliography, i.e., PDMS (Bélanger and Marois, 2001; Llobera et al., 2009), air (Llobera et al., 2009), blood (Roggan et al., 1995; Tuchin, 2007) and the microvessel (Roggan et al., 1995; Tuchin, 2007). For consistency with experimental data, ray tracing simulations were performed considering the light source and detector connected to 3 m long optical fibers (cladding diameter 230 μm; core diameter 200 μm; numerical aperture 0.22). Total irradiance values were acquired at 700 nm for opto-mechanical analysis.

Photonic microsystems were fabricated by soft-lithography in PDMS (Xia and Whitesides, 1998) (for fabrication details see Supplementary Information S8 and Figure 2A).

### Optical Measurements

Opto-chemical and opto-mechanical measurements were performed using the broadband halogen lamp HL-2000-FHSA (Ocean Optics, USA) as light source and an USB-2000 microspectrometer (spectral resolution 3 nm; dynamic range 25 dB; spectral range 350–1,000 nm; Ocean Optics, USA) as detector connected to multimode optical fibers (200 μm core/230 μm cladding multimode; NA = 0.22; Thorlabs, Dachau, Germany). Spectra Suite software (Ocean Optics, USA) was used in the measurements for data acquisition. Integration time was fixed in accordance with the experiment.

### *In vitro* Pressure Myography

Pressure myography (111P, Danish Myo Technology A/S, Denmark) was chosen as validation method for determination of the mechanical performance of the microvasculature *in vitro*. Rat small mesenteric arteries were used as model. Arterial isolation, implementation in the myograph and protocols for structural and functional analysis followed the protocol reported elsewhere (Rodríguez-Rodríguez et al., 2009; Ogalla et al., 2015) and detailed in Supplementary Information S9. Arterial distension was determined as changes in the microvessel caliber. Briefly, intraluminal pressure was increased sequentially from 0 to 120 mmHg in regular steps of 10 mmHg using the pressure myography system. Arterial diameter at each applied pressure was determined, as follows. Arterial caliber was recorded by video imaging (CCD camera and frame grabber coupled to PC acquisition system with MyoVIEW software) and the diameter was determined through manual analysis, i.e., frame by frame analysis every 5 s.

The calibration of microsystem performance was conducted as described below. First, the microsystem was implemented manually. The lightguide-cantilever was manually deflected with tweezers and the artery was positioned at the very end of the cantilever. A cannula was used to clamp the optical fibers inserted in the self-alignment elements thus avoiding mechanical stresses that may affect the measurement. Once clamped, intraluminal pressure was increased as before and optical losses in the photonic microsystem were compared to videorecords. Comparing microsystem and myograph data, it was possible to associate optical losses with arterial diameter changes at each intraluminal pressure under study.

### Intravital Microscopy

Intravital microscopy was selected as the validation method for the *in vivo* analysis of the rat microvasculature (Nyvad et al., 2017). A short segment of the intestine and the mesentery of anesthetized animals was pulled out by a protocol reported elsewhere (Dam et al., 2014) (Supplementary Information S10). Arterial distension was determined as a change in the artery caliber. Also, derived from this data, stiffness and general viscoelastic properties were calculated. Functional tests involved vasoconstriction with noradrenaline (NA; 5 μM) and relaxation with ACh (5 μM). The microsystem was implemented in the artery as previously detailed. Videomicroscope and microsystem recordings were obtained simultaneously in the same arterial region. Fat was cleaned around the whole vessel in the region under study and the microsystem was implemented. The integration time was set between 100 and 250 ms, depending on the experiment. Transmittance values at a single wavelength were considered, i.e., at 700 nm, where the absorption of the tissue was low.

### Data Analysis and Statistics

All results are expressed as mean ± SD, unless otherwise stated. *P* < 0.05 was considered significant and *n* refers to the number of animals. Functional responses were expressed as a percentage of the previous tone generated by Phe. Statistical significance was obtained by *t*-test, when comparing two groups. Data analysis was carried out using GraphPad Prism Software 5.0 (San Diego, CA, USA).

## Conclusions

The evaluation of bidirectional interaction between small arteries and surrounding tissues *in vivo* is possible with the highly-integrated and miniaturized all-polymer photonic microsystem characterized in the current study. The microsystem reports simultaneously on arterial stiffness, distension, vasomotion and heartbeat through an unprecedented opto-mechanical sensing mechanism named optical myography. The optical microsystem can operate in three configurations, according to the surrounding tissue: (i) arteries isolated from the animal (*in vitro*) where the surrounding tissue has been partially removed (Figure 3), (ii) non-isolated arteries (*in vivo*) where the surrounding tissue has been partially removed (Figure 4), and (iii) intact arteries, non-isolated from the animal (*in vivo*) and integrally preserving the surrounding tissue (Figures 5A,B). In the analysis of arterial segments (*in vitro*) or arteries *in vivo* partially isolated from the surrounding tissue, this technology complements myography without requiring imaging or tracking systems, and improve their performance in terms of simplicity, cost, limit of detection, linear range, sensitivity and repeatability. In addition, the photonic microsystem allows the analysis of intact arteries, without removal of surrounding tissues and preserving their integrity and function. In short-term, arterial vasoconstriction and relaxation in the microvasculature of living rats modify blood vessel distension and stiffness, as well as vasomotion, all of which is simultaneously recorded with the microsystem. The *in vivo* analysis of intact microvessels containing fat and connective surrounding tissues demonstrates that, apart from structural, these tissues have an active function being responsible of an important pre-constriction of the vessel. We envisage the current technology as one of the most promising alternatives to study bidirectional interaction between arteries, surrounding tissues and organs and their alterations leading to cardiovascular diseases.

## Data Availability

All datasets generated for this study are included in the manuscript and/or the Supplementary Files.

## Ethics Statement

The protocol for animal handling and experimentation was in agreement with the European Union European Community guidelines for the ethical treatment of animals (UE Directive of 2010; 2010/63/UE) and was approved by the local Ethical Committee for Animal Research of Aarhus University.

## Author Contributions

RR-R performed the *in vitro* and *in vivo* assays and assisted XM-B in the interpretation of data and writing of the paper. TA performed the ray tracing simulations and assisted XM-B in the interpretation of data and writing of the paper. JP contributed in the design of the microsystem, performed the mechanical simulations, and assisted XM-B in the interpretation of data and writing of the paper. US contributed in *in vitro* validation of the microsystem and assisted XM-B in the interpretation of data and writing of the paper. VM contributed on the *in vivo* validation of the microsystem and assisted XM-B in the interpretation of data and writing of the paper. AL designed and fabricated the microsystem and assisted XM-B in the interpretation of data and writing of the paper. XM-B contributed in the design, fabrication, *in vitro* and *in vivo* validation of the microsystem and wrote the paper, in consultation with all authors.

### Conflict of Interest Statement

The authors declare that the research was conducted in the absence of any commercial or financial relationships that could be construed as a potential conflict of interest.

## Footnotes

^1^https://www.who.int/news-room/fact-sheets/detail/cardiovascular-diseases-(cvds) (accessed December 20, 2018).
