# Supplementary material for: Ultrasensitive Photonic Microsystem Enabling Sub-micrometric Monitoring of Arterial Oscillations for Advanced Cardiovascular Studies
Source: Front Physiol. 2019 Jul 23;10:940. doi: 10.3389/fphys.2019.00940 (PMC6664303; doi:10.3389/fphys.2019.00940)
Supplement: Supplementary file 1 [file Data_Sheet_1.PDF]

Supplementary Material

Video S1. Video illustrating the mechanical changes of the lightguide-cantilever after increasing the applied pressure in the cylindrical structure (emulating a microvessel) from 0 mmHg to 120 mm Hg (first frame of the video here; full video attached as an independent avi file: video SI.1).

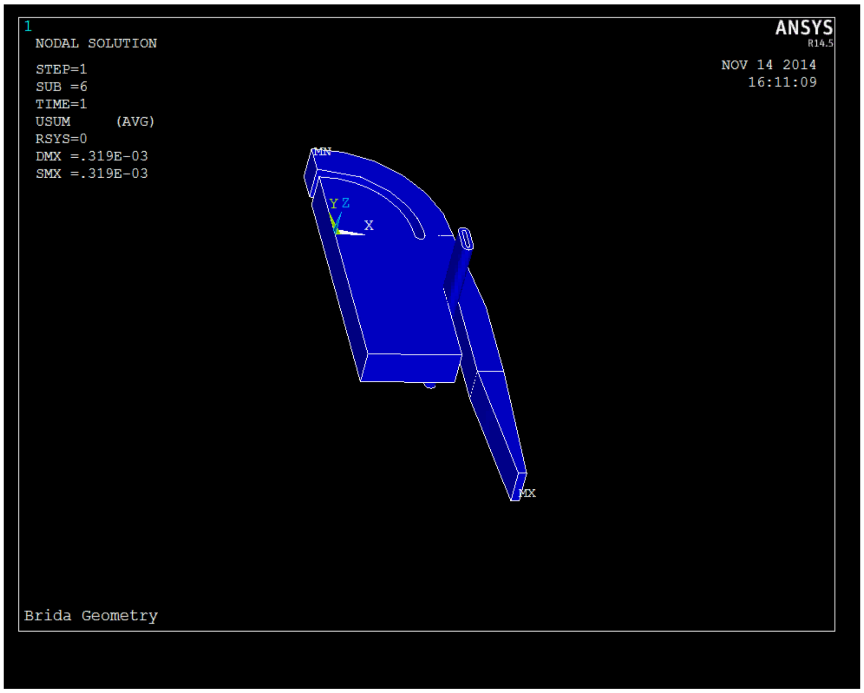

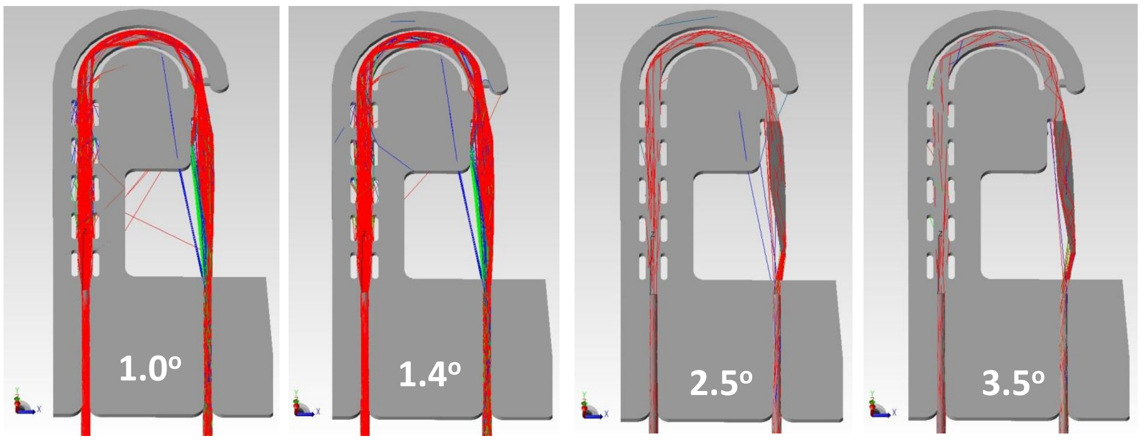

Figure S2. Ray tracing simulation images illustrating the rays reaching the detector at 1.0º, 1.4º, 2.5º and 3.5º deflection angles. Only light rays reaching the detector are illustrated. The color of the ray illustrates its intensity, being the red more intense than blue and green, which are the less intense of all of them.

S3. Details on ray-tracing simulations for in-plane air cavities design.

Regarding the elastomeric photonic microsystem design, a U-shaped architecture (generating a U-shaped PDMS lightguide) was chosen for light confinement and guidance from light inlet to the lightguide-cantilever. For simplification, the U-shaped set of air cavities was considered to be composed of two independent sets: a set of straight air cavities and two curved cavities (inner and external curved cavities). Straight cavities were designed as a combination of multiple cavities (instead of two parallel cavities) to ensure light guidance without compromising mechanical properties of the dual sensing microsystem. In opposition, air cavities in the curvature were continuous to maximize light guidance. Each set of cavities was analyzed individually using the ray-tracing simulation software Trace Pro (Lambda Research, Littleton, MA, USA). Five configurations containing different sets of cavities (i.e. a full set of straight and curved cavities, FSM, only curved cavities, CM, only the external curved cavities, ECM, only straight cavities, SM, or without air cavities, WM) were designed and evaluated. Optical properties of each element in the model were adjusted according to the constituent material, that is polydimethylsiloxane, PDMS (n_{PDMS} = 1.42) or air (n_{air} = 1). For consistency with experimental data, light source and detector were considered to be connected to 3 m long optical fibers with the same featuring than those used experimentally (cladding diameter 230 µm; core diameter 200 µm; numerical aperture 0.22). In each optical simulation, a minimum of 40000 rays were analyzed.

Most relevant simulation results are summarized in Figure SI.1. In Figure SI.1A, optical path and reflections of light rays reaching the detector are illustrated by each configuration under study. Rays color in the figure indicated their intensity, being the red ones much more intense than the blue ones. Irradiance maps for signal (light rays collected in the detector after confinement and guidance along the lightguide-cantilever, the sensing element) and signal + noise (total light rays collected in the detector) and are also included in this figure. Additionally, total irradiance magnitude (in percentage terms, considering an initial flux of 1 W) and SNR corresponding to each configuration are plotted in Figure SI.1B and SI.1C, respectively. According to this, the integration of the full set of cavities (straight and curved cavities) only ensured the collection of 1.1% of the total flux, which was reduced by half when straight cavities were removed from the structure (0.6% irradiance), and by ten when removing curved (0.15%) or the full set of cavities (0.15%). Thus, although both straight and curved cavities contributed to light guidance, curved cavities, and particularly external curved cavity architecture, was fundamental to ensure light guidance in the sensor. Conversely, straight cavities played a crucial role in the reduction of the measurement noise: structures without straight cavities presented low SNR ratios (15 when still containing curved cavities or around 2 without cavities), whereas microsystems only containing straight cavities presented a SNR of 31, very close to that obtained when incorporating the full set of cavities (36). Thus, in terms of cavities, microsystems required straight (to reduce noise in the measurement) and curved (to enhance light guidance and collection) cavities to ensure optimal performance of the system.

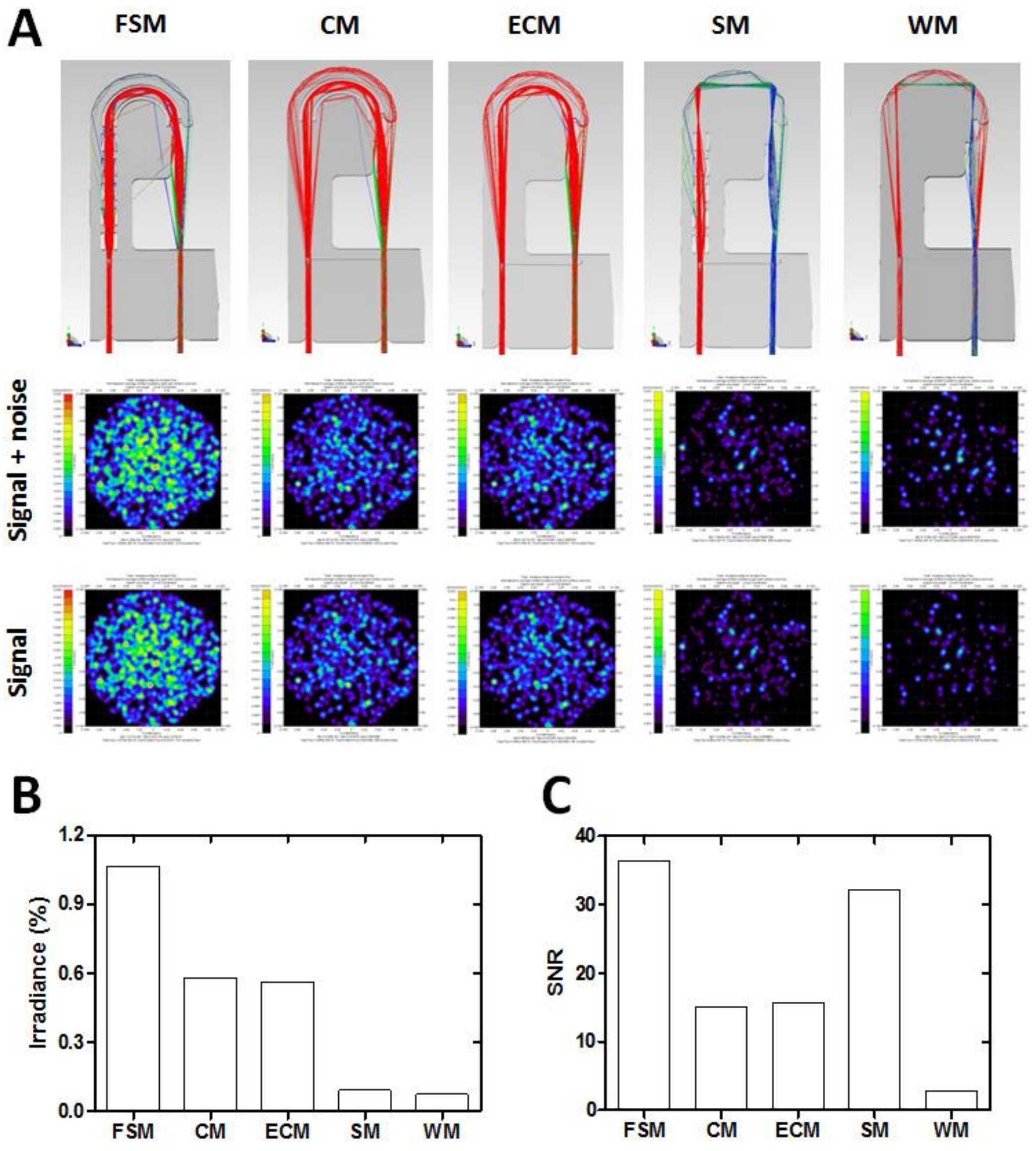

Figure S3 Air cavities performance. (A) Ray tracing illustration for the five microsystem configurations containing the full set of air cavities (FMS), curved cavities (CM), the external curved cavities (ECM), straight cavities (SM) or without cavities (WM). Only rays reaching the detector are illustrated. Red rays are more intense than blue ones. Also the irradiance maps for signal + noise (total rays collected in the detector) and signal (rays collected in the detector after confinement and guidance in the lightguide-cantilever) are included. Representation of (B) the total irradiance (in percentage terms) and (C) signal-to-noise ratio (SNR) for each microsystem architecture under study.

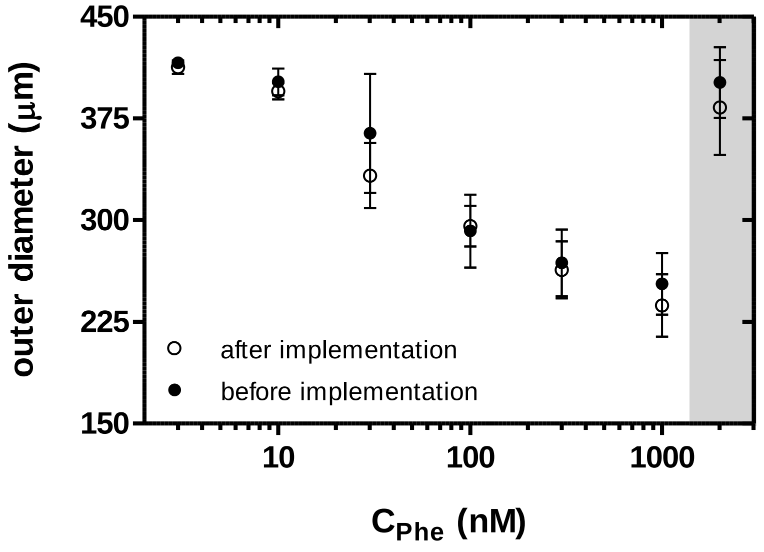

Figure S4. Outer diameter magnitude (from videomicroscope) in response to increasing concentrations of Phe before and after the implementation of the microsystem (n=3).

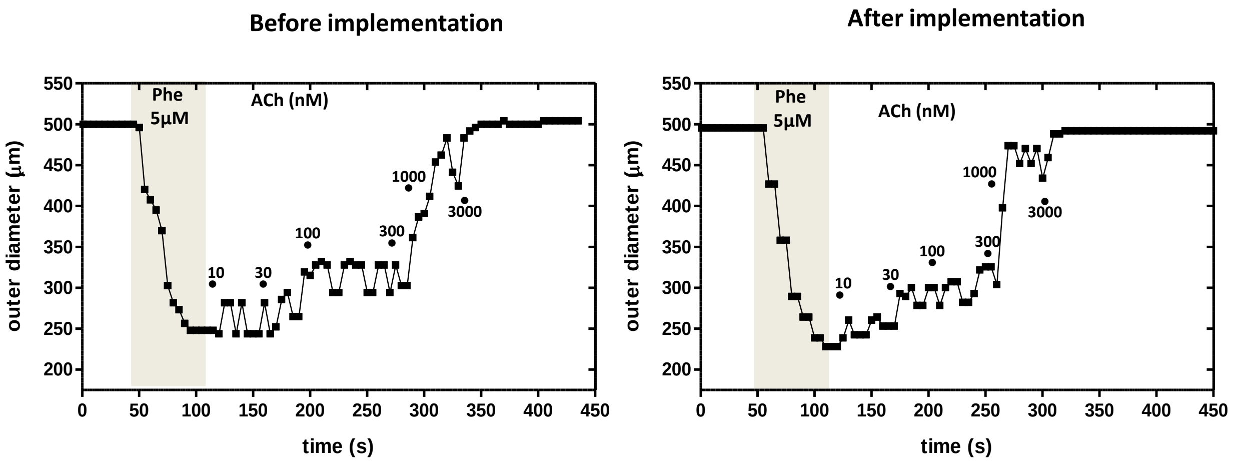

Figure S5. Variation of the outer diameter magnitude (from videomicroscope) in response to increasing concentrations of ACh, before and after the implementation of the photonic microsystem.

Video S6. In vivo video recording of the deflection of the lightguide-cantilever in response to heartbeat. For better understanding, the first frame and the schematic of the first frame is here shown. In the schematic, the lightguide-cantilever is in purple, the microsystem is in grey and the artery is in red (full video attached as an independent avi file: video S8).

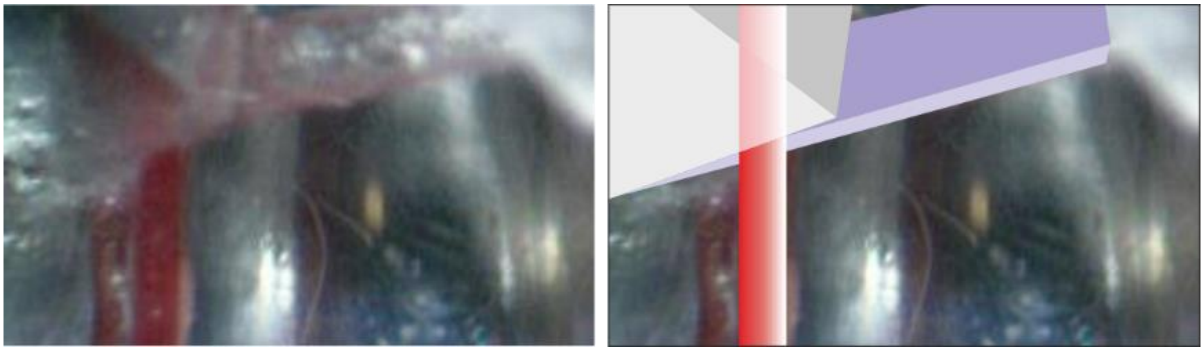

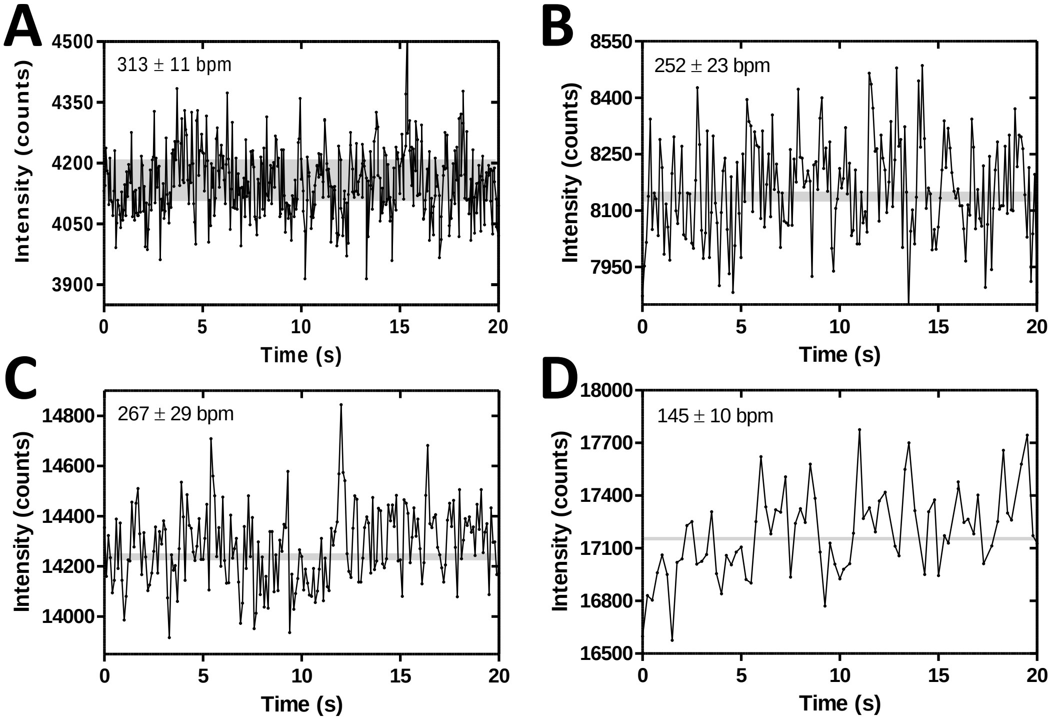

Figure S7. Representation of light intensity variation with time in response to deflection of the lightguide-cantilever due to heartbeat at integration times of (A) 50 ms, (B) 100 ms, (C) 200 ms and (D) 250 ms. Electrical noise is illustrated in grey in the plot. The average heart rate calculated as the number of oscillations (beats) per minute is included inset.

S8. Details on the microfabrication protocol.

Photonic microsystems were fabricated by soft-lithography(Xia and Whitesides, 1998) in PDMS. The protocol involved the fabrication of a two-level master(Llobera et al., 2009; Muñoz-Berbel et al., 2013) as follows. A 125 µm SU-8 (MicroChem Corporation, Newton, MA, USA) layer was deposited (by spinning) on a 500 µm-thick silicon/silicon oxide wafer. After a baking of 1h (starting with a temperature ramp from 65 ºC -to 95 ºC), it was exposed to UV radiation (total energy = 230 mJ/cm^2) through a mask to define optical and mechanical elements of 125 µm height. A post exposure bake (65 ºC - 95 ºC ramp; 15 min) was performed to minimize stresses and to avoid cracks in the polymerized material. A second 125 µm thick SU-8 layer was then deposited and exposed in alignment with the first one to define the elements that required additional height, for example the frames of the individual moulds. The final master was obtained after development of structures by immersion in propylene glycol methyl ether acetate solution (PGMEA, MicroChem Corporation, Newton, MA, USA) followed by a hard bake at 120 ºC to minimize stresses and to partially heal cracks or defects.

The two-level master was replicated with PDMS (Sylgard 184 elastomer kit, Dow Corning, Midland, MI, USA). PDMS was prepared as described by the supplier (10:1 elastomer:curing agent) and carefully dispensed inside the frames, which prevented the spreading of the pre-polymer solution. Once deposited, the PDMS was cured for 20 min at 80 ºC. Final microsystems were obtained after solvent-assisted alignment and bonding of two PDMS layers, i.e. a first one comprising the functional elements and a second one inclosing well-defined air-pockets in the inner layer. To that end, both PDMS layers were exposed to oxygen plasma (500 W, 18 s) and aligned using a lubricant (i.e. 96% ethanol) to slow down bonding. Permanent bonding required 20 min at 60 ºC.

S9. Arterial isolation protocol.

Rat mesentery was collected from male adult Wistar rats (200 g - 250 g body weight) and placed in cold modified Krebs-Henseleit Solution (KHS) (composition: 118 mM NaCl, 4.75 mM KCl, 25 mM $NaHCO_3$, 1.2 mM $MgSO_4$, 1.8 mM $CaCl_2$, 1.18 mM KH_2PO_4 and 11 mM glucose). KHS (pH 7.4) was gassed with 5 % CO_2 in O_2. Second or third order branch small mesenteric arteries (outer diameter = 150 µm -300 µm) were selected and isolated after removal of surrounding connective tissue.

Isolated arterial segments were mounted in the myograph by cannulating each arterial end with glass micropipettes and securing them with nylon suture. Intraluminal pressure was then raised to 120 mmHg to test for potential leakage and to adjust the standard distance between cannulas.

S10. Protocol for in vivo isolation and analysis of small mesenteric arteries.

Male adult Wistar rats (250 g – 300 g) were anesthetized by subcutaneous injection of ketamine (33 mg/100 g; Intervet International, Holland) and xylazine (7.5 mg/100 g; Intervet International, Holland) and placed on a heating plate (37 ºC). After lateral laparotomy, a short segment (1.5 cm - 2 cm) of the intestine and mesentery was carefully pulled out. A single branch of a 2nd or 3rd order mesenteric artery was selected and approximately 50 % of the surrounding fat was dissected from the top of the segment to enable visualization. Arterial diameter was captured with a USB CCD Monochrome Camera (DMK 41 AU02, Imaging Source, Germany) attached to the microscope (Motic PSM-1000) and processed using DMT Vessel Acquisition Suite software (Danish Myo Technology A/S, Denmark). The selected arterial segment was immersed in physiological saline solution (PSS) containing 10 mM HEPES (HEPES-PSS) and bubbled with 5 % CO_2 in N_2. During experiments, extracted intestine and mesentery were kept wet using bandages soaked in HEPES-PSS.

References

Llobera, A., Cadarso, V. J., Zinoviev, K., Dominguez, C., Buttgenbach, S., Vila, J., et al. (2009). Poly(Dimethylsiloxane) Waveguide Cantilevers for Optomechanical Sensing. *IEEE Photonics Technol. Lett.* 21, 79–81. doi:10.1109/LPT.2008.2008659.

Muñoz-Berbel, X., Rodríguez-Rodríguez, R., Vigués, N., Demming, S., Mas, J., Büttgenbach, S., et al. (2013). Monolithically integrated biophotonic lab-on-a-chip for cell culture and simultaneous pH monitoring. *Lab Chip* 13, 4239–47. doi:10.1039/c3lc50746g.

Xia, Y., and Whitesides, G. M. (1998). Soft Lithography. *Angew. Chemie Int. Ed.* 37, 550–575. doi:10.1002/(SICI)1521-3773(19980316)37:5<550::AID-ANIE550>3.0.CO;2-G.
